# Picking strategies in games of cooperation

**DOI:** 10.1073/pnas.2319925121

**Published:** 2025-06-16

**Authors:** Julian García, Arne Traulsen

**Affiliations:** ^a^Department of Data Science and AI, Monash University, Melbourne, VIC 3800, Australia; ^b^Department of Theoretical Biology,Max Planck Institute for Evolutionary Biology, Plön 24306, Germany

**Keywords:** cooperation, game theory, dynamics, agents, evolution

## Abstract

Evolutionary game theory (EGT) has been pivotal in the study of cooperation, offering formal models that account for how cooperation may arise in groups of selfish, but simple agents. This is done by inspecting the complex dynamics arising from simple interactions between a few strategies in a large population. As such, the strategies at stake are typically hand-picked by the modeler, resulting in a system with many more individuals in the population than strategies available to them. In the presence of noise and with multiple equilibria, the choice of strategies can considerably alter the emergent dynamics. As a result, model outcomes may not be robust to how the strategy set is chosen, sometimes misrepresenting the conditions required for cooperation to emerge. We propose three principles that can lead to a more systematic choice of the strategies in EGT models of cooperation. These are the inclusion of all computationally equivalent strategies; explicit microeconomic models of interactions, and a connection between stylized facts and model assumptions. Further, we argue that new methods arising in AI may offer a promising path toward richer models. These richer models can push the field of cooperation forward together with the principles described above. At the same time, AI may benefit from connecting to the more abstract models of EGT. We provide and discuss examples to substantiate these claims.

The evolution of cooperation has been a popular subject of interdisciplinary research for decades (1, 2). As a puzzle identified already by Darwin (3), it has attracted the attention of Biologists, Social Scientists, Mathematicians, and those interested in Complex Systems, including Physicists and Computer scientists (4).

Today, the evolution of cooperation can no longer be considered a puzzle. The foundational mechanisms behind the evolution of cooperation are well understood in principle. But open questions remain in explaining stylized empirical findings from an evolutionary perspective. Here, we argue for three principles that can help ensure that new models of cooperation are relevant in pushing the field forward. These principles ensure that models are robust, and discourage models with ad hoc mechanisms, often disconnected from the literature.

We further relate these principles to new work on cooperation arising in the field of AI. We argue that new approaches in AI can enrich the literature of cooperation and provide a path to rich and robust models of cooperation.

In its simplest form, cooperation means paying an individual cost in order to help somebody else (5). This is typically studied using a prisoner’s dilemma, where individuals have the choice to i) cooperate, assuming an individual cost to bestow a benefit on their coplayer or ii) defect, in which case no cost is paid, but there is a potential benefit in exploiting others that cooperate. The dominant strategy of this game is defection, as defection always yields a higher payoff. However, everyone is better off if everyone would be cooperating—hence the dilemma.

In the field of cooperation, the vast majority of research revolves around proposing and analyzing mechanisms for cooperation. These mechanisms respond to plausible hypothesis and tend to shift interests in a way that makes cooperation sustainable in equilibrium (5). A proximate explanation for human cooperation may be the existence of social preferences. Why these social preferences may have evolved—for example due to population structure—is the ultimate explanation that evolutionary models are often seeking.

Arguably, the most important formal tool in this field is evolutionary game theory (EGT) (6–8). This tool itself has grown and developed with the study cooperation. Originally based on static solution concepts rooted in classical game theory (6), EGT now encompasses a suite of deterministic dynamics (8), stochastic dynamics (9) and computational and simulation techniques (10, 11).

On the basis of EGT, a lot of progress has been made, from considering the evolution of cooperation an open puzzle (12), to having found many potential solutions to this problem (13) and to a discussion of the relationships between them (1, 14, 15). Contemporary research in cooperation focuses less on finding new mechanisms and more on understanding how mechanisms can interact to explain stylized behavioral findings.

Evolutionary game theorists try to develop and understand mechanisms in which individual optimization could still lead to the evolution of cooperation, despite defection leading to a higher immediate payoff. This can only happen if there is a mechanism that promotes cooperation (1). While there is long-standing debate about the relation between different mechanisms (14, 16), the goal is typically to find plausible explanations for cooperation: for example, social insect societies cooperate based on their genetic architecture or humans cooperate through various mechanisms of reciprocity (17).

Often the goal is to understand the system analytically at least in some limit, for example, weak selection (9), low mutation rates (26) or in the limit of large populations (8). These limits are often of no direct real world relevance, but they allow generalization between systems (8), guiding the understanding of a wider parameter regime.

The complex role of spatial structure in such systems is appreciated in EGT (27–32), but many models focus on strategic choice without looking at the intricacies coming with spatial structure.

The de novo evolution of cooperative solutions is usually a more challenging problem than stabilizing cooperative solutions. But in both cases, the choice of the strategy set is crucial: cooperation may evolve for some strategy set, but if mutations create strategies outside this set, it may break down again. If we engineer a robust strategy set, we also need to think of such possible deviations: is my agent interacting with agents of a different internal architecture that allows for other deviations? Stabilization is relatively straightforward if we have a grasp on all possible invading strategies, but neither in evolutionary biology nor in computer science that may be the case: in EGT, we focus on few strategies and often forget that any real biological system has myriads of ways to deviate from these. Interacting artificial agents may ultimately be subject to a clear external regulation, for example for the interaction between self-driving cars—but the current systems in which interactions between artificial agents and humans occur are less controllable and it is unlikely that algorithmic details of commercial agents become fully transparent. One recurring issue in this context is the choice of strategy sets, which we discuss here.

In the following, we first introduce briefly the typical way to model the problem of cooperation in EGT, both in terms of deterministic and of stochastic models. In particular, we focus on the way that the strategies in a model are typically chosen. This will give rise to three principles for choosing the strategy space.

## 1. The Toolset of EGT

### 1.1. Static Solution Concepts.

EGT was initiated more than 50 y ago by Maynard Smith and Price (36). They set out to explain the observation of limited war strategies between animals fighting. They picked different strategies and showed how strategies that limit their aggression can be most successful, in the sense that when they are established they cannot be invaded by mutants. EGT was then established as a tool to show how evolution was aligned with behaviors observed in evolutionary ecology, such as food sharing (37), predator inspection (38), cyclic competition between mating types (39), Mafia behavior in brood parasites (40), or explanations for the reciprocal instincts that permeate the social interactions in humans (17).

In all these instances, EGT was invoked to provide an explanation of why certain behaviors observed in nature are compatible with evolution. In many cases, it is enough to work with the payoffs in these games to identify evolutionary stable strategies (ESS) that can be expected to be observed (6).

The ESS concept is a refinement of the Nash equilibrium (7). It can single out outcomes in cases where multiple Nash equilibria exist, but it can still lead to several possible predictions with multiple ESS, or no predictions when an ESS does not exist. This kind of approach makes statements on possible evolutionarily stable states, i.e., sets of strategies that cannot be invaded once a population has reached them. But it is silent about the path to get there.

### 1.2. Deterministic Dynamics.

Evolutionary dynamics in the modern sense was introduced to game theory in ref. 41 and 42. The standard model is the replicator dynamics, where the change in abundance *x*_*i*_ of a strategy *i* is assumed to be

[1] $$\begin{array}{c} \frac{d}{\mathrm{dt}}x_{i}=x_{i}\left(f_{i}-\left\langle f\right\rangle \right), \end{array}$$

where *f*_*i*_ is the fitness of strategy *i*, $\left\langle f\right\rangle =\sum_{j=1}^{n}f_{j}x_{j}$ is the average fitness in the population and *n* is the number of strategies. Here, dynamics is deterministic and population size is implicitly assumed to be infinite.

While we often focus on the analysis of fixed points of this dynamics, sometimes these equations can be solved analytically or constants of motion (or Lyapunov functions) can be identified. Some very insightful general statements can be made: for example, strategies that are always inferior to other strategies will never be present in an evolutionary stable equilibrium (8). This allows to eliminate such dominated strategies and can simplify the analysis tremendously.

There are also general results about other dynamics proven to be asymptotically equivalent, and results that connect static solution concepts to these dynamics. Importantly, in general, these dynamics do not specify the path of evolution, and strongly depend on initial conditions, which is sometimes limiting in making predictions or providing explanations.

### 1.3. Stochastic Models of Finite Populations.

The most popular model for stochastic evolutionary game dynamics is the Moran process (9), where a finite population of fixed size *N* is considered in which payoffs *π*_*i*_ of each individual type *i* arise from interactions with a representative sample of the population. In each time step, one individual is selected proportional to fitness *f* (an increasing function of payoff *π* (43, 44), e.g., f= exp(βπ), where *β* is the intensity of selection). This individual produces identical offspring (with probability 1−μ) or a mutant with a random strategy (with probability *μ*). Finally, the offspring replaces a randomly chosen individual.

This extension of EGT toward stochastic models (9) has greatly enriched the field and led to several new models and approaches. It has allowed to make new connections to population genetics (45, 46), develop new mathematical methods for structured populations (47, 48) and led to a heated debate on the relationship between EGT and kin selection theory (16). However, at the same time, this new modeling approach causes new issues in the relationship between EGT and classical game theory. For example, dominated strategies can safely be discarded in classical game theory and in the traditional models of EGT based on the replicator dynamics, as they have no important influence (8). Under weak selection (*β* ≪ 1 above), however, dominated strategies can strongly affect the results, either by changing equilibrium abundances or by changing the course of evolution through neutral paths (49). If such complications would arise only in the regime of weak selection, they would not pose a serious issue, as the regime of classical EGT is very far from this (50). However, there are examples where even under strong selection, evolutionary games show different results when neutral strategies are included or where the precise definition of strong selection becomes important (43, 51).

### 1.4. The Strategy Space.

Evolutionary models aim to find explanations for behaviors or traits we observe. However, ideally such models should not only explain what is there but also why other traits are not there. Moreover, traits that appear in low abundance can still be crucial for evolutionary dynamics. This implies that such traits should be included in evolutionary models, even if they are not observed in the end. Given these complications, in which way should we choose the strategy sets we consider? Here, we propose to define strategy sets not in an arbitrary fashion or based on intuitive arguments, but to use a systematic approach that typically leads to a larger number of strategies. Then, evolution should decide what is relevant. Given the large strategy set, a more detailed analytical analysis may only be possible for a smaller subset of strategies that is identified by this numerical approach.

In the simplest case, game theoretical models have only two strategies, e.g., cooperation and defection. Both strategies have the same (very limited!) complexity and they represent both the presence of a behavior (cooperation) and its absence (defection). Once we start building upon this to include more complex behaviors, complications can arise: If we add memory, should we allow all possible ways to use this memory? If we want to explain pro-social punishment, is it necessary to include the possibility of antisocial punishment from the start? It is hard to answer these questions. A possible solution is to let evolution decide and define strategy sets based on more systematic considerations. We thus suggest three principles for the choice of the strategy set:

1. Check that all strategies are computationally equivalent and unbiased.
2. Develop a microeconomic model for the interactions.
3. Establish a connection to stylized facts.

We next discuss these principles in detail, and give examples substantiating why they are important for the progress of the field. For each principle, we start the discussion with an example, and further develop the ideas behind the principle.

## 2. Three Principles for Choosing the Strategy Space

### 2.1. Principle 1: Unbiased and Computationally Equivalent Strategies.

#### A simple model of repetition in the prisoner’s dilemma.

Repeated games encapsulate the principle of direct reciprocity, whereby agents can trade the cost of cooperation for the future benefits of reciprocation: “you scratch my back and I’ll scratch yours” (17, 52). To illustrate the approach of standard EGT, we use the repeated prisoner’s Dilemma, assuming that agents engage in repeated interactions. Therefore, strategies account for a time element and are more complex than simply cooperate and defect. A strategy for a repeated game specifies which action to play given the history of play. In a game that is repeated with probability *δ*, the payoff $\Pi_{\mathrm{AB}}$ of *A* when it faces *B* is given by

[2] $$\begin{array}{c} \Pi_{\mathrm{AB}}=\sum_{i=0}^{\infty }\delta^{i}\pi_{\mathrm{AB}}^{i}, \end{array}$$

where $\pi_{\mathrm{AB}}^{i}$ is the pay-off in the *i*-th round of the game. The one shot game we are interested in is a prisoner’s dilemma with the pay-off matrix $(\begin{array}{cc} R & S\\ T & P \end{array})$, with T>R>P>S and $R>\frac{T+P}{2}$. The expected number of rounds is $\sum_{i=0}^{\infty }\delta^{i}=\frac{1}{1-\delta }$. It is convenient to normalize the pay-off of the repeated game multiplying by 1−δ, making the magnitudes of the repeated and the single-shot game comparable.

The starting point in traditional model is to define strategies and the corresponding game between them. We first focus on three strategies, *AllC* (always cooperates), *TFT* (starts to cooperate and then copies the opponent), and *AllD* (always defects). The corresponding normalized payoff matrix




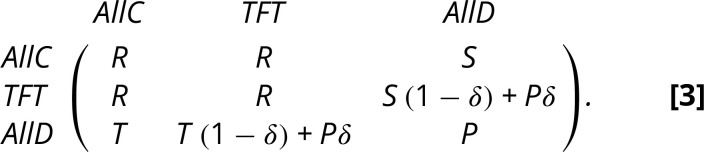




**Fig. 1.**
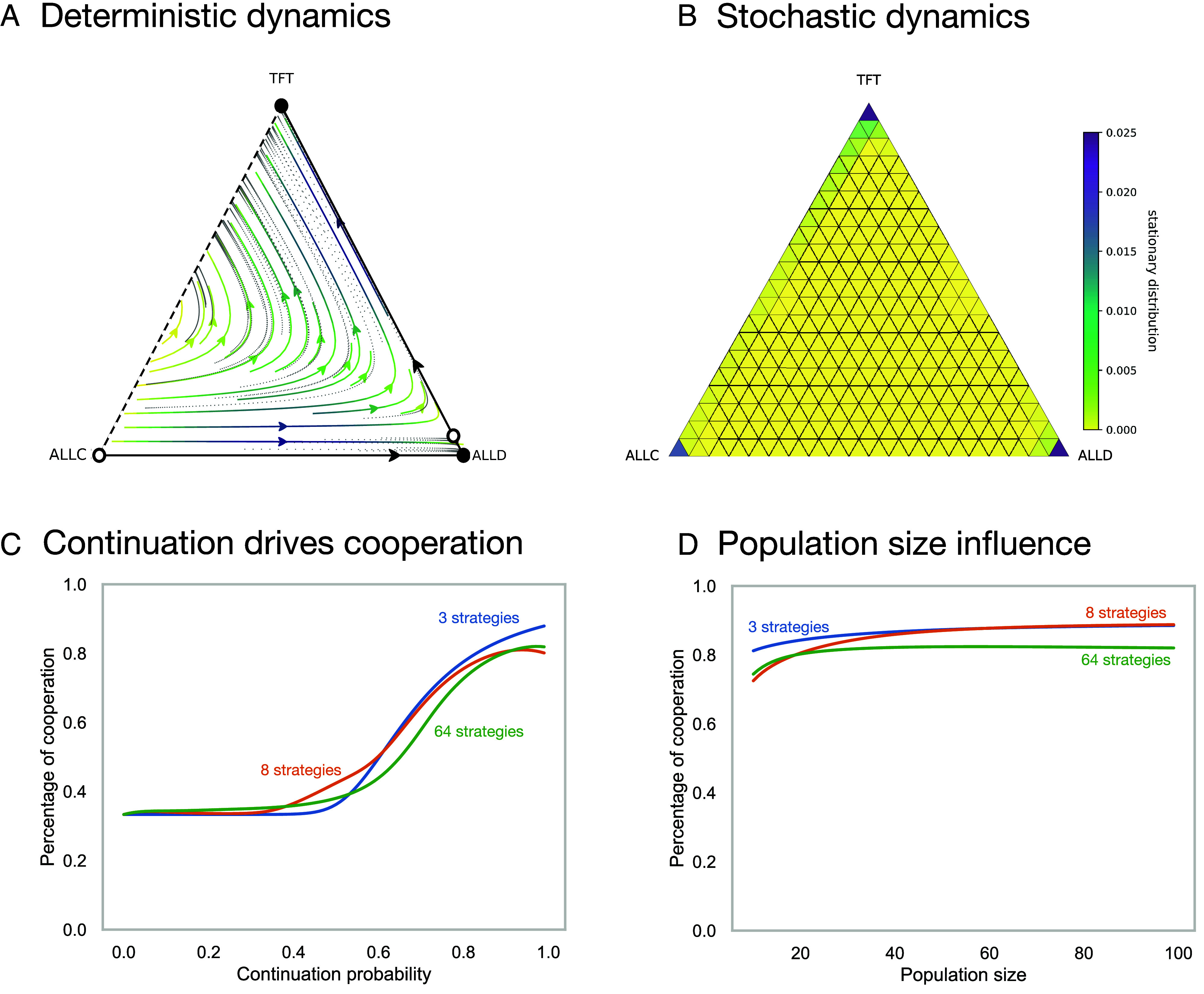
Strategy spaces in the repeated prisoner’s dilemma. Panel *A* shows the deterministic dynamics arising from the replicator equation **1**. Panel *B* shows the long-term behavior of the stochastic dynamics arising from an imitation process with exponential payoff to fitness mapping. In the stochastic process, the population size is *N* = 20, intensity of selection *β* = 1.0, and exploration parameter—or mutation probability—$\mu =10^{-3}$. With the demographic noise introduced in the stochastic process, we see that the population spends a significant amount of time in the neutral edge between reciprocal and indiscriminate cooperation (TFT and ALLC), such that all players cooperate. However, neutral drift may increase the number of AllC players, which can easily be invaded by AllD. Panels *C* and *D* show the average percentage of cooperation reached in the long-term for three different choices of strategy set in an imitation process. Cooperation is overestimated with the biased strategy set of three strategies. Panel *C* shows how *δ* influences the level of cooperation for *N* = 20. Panel *D* shows how population size *N* affects cooperation for *δ* = 0.9 (Game parameters *R* = 3, *S* = 0, *P* = 1, *T* = 4).

In a standard prisoner’s dilemma between *AllC* and *AllD*, defection is the only Nash equilibrium. In contrast, this game of three strategies has two pure Nash equilibria: AllD and TFT, as two players playing *TFT* have no incentive to deviate when *δ* is large enough. The immediate consequence of this is that with reciprocity at play, cooperation can now be sustained in equilibrium—in the static sense. However, also defection can be sustained. To decide which equilibrium is approached, one can switch to a more sophisticated approach. Historically, Axelrod and Hamilton (17) famously introduced a round-robin tournament where scientists could submit strategies, leading to the success and the subsequent popularity of the TFT strategy. However, it should be noted that this success is highly environment dependent: As TFT does not win in any pairwise contest (it never defects more often than the opponent), other tournament setups would have led to very different outcomes. A theoretical approach based on evolutionary dynamics instead is illustrated for deterministic and stochastic dynamics in Fig. 1.

Using the replicator dynamics described above sheds light on the actual dynamics of evolutionary competition. While both TFT and ALLD are stable equilibria of the dynamics, the starting point of the dynamics can lead to one or the other solution (Fig. 1*A*).

The stochastic dynamics is examined using an imitation process (53). Here, the neutral dynamics between ALLC and TFT becomes important. As ALLC and TFT get the same payoff, stochastic noise is crucial. When the noise renders the number of AllC players sufficiently high, AllD players can invade and defection spreads. Thus, in the long run, it becomes crucial how TFT can invade AllD again.

A similar system is studied by Imhof et al. (54), who introduced an additional complexity cost for Tit-for-Tat. This leads to cycles, as the neutral edge disappears and indiscriminate cooperation can take over the costly reciprocal strategy. This model is a nice illustration of the cycles that emerge in such a stochastic system, but it would be problematic to use it to explain the evolution of cooperation, as it gives an unfair advantage to cooperation: under weak selection, the cooperative strategies (TFT and AllC) are more abundant than the single defector strategy (AllD).

#### All strategies of a given memory size.

The three-strategies space with ALLC, ALLD, and TFT is biased. All strategies consider the opponent’s last move, but not all such strategies are present. Including all such strategies has a modest but noticeable effect in the outcome of the model. In particular, cooperation is overestimated with a small biased strategy space. This is shown in Fig. 1.

Including all other strategies that are conditioned on the opponent’s last move still leads to the evolution of cooperation based on reciprocity—but interestingly, even strategies that would normally be discarded, e.g., to cooperate once and then always defect, can have a similar high abundance as AllD. For very strong selection, the same two Nash equilibria TFT and AllD emerge as the most abundant strategies, but for weaker selection other strategies matter (55).

For longer memory, the space of possibilities grows rapidly and a modeling approach where all strategies are always present becomes problematic. An alternative approach is to define only the way that strategies are encoded and open the memory length and thus the space of strategies entirely (49, 56)—but such an approach can only be handled computationally.

The argument here is not that the simple model with 3 strategies is not appropriate. The argument is the need to produce a robustness check arising from a set that is not biased. We believe this principle should apply generally in models addressing the evolution of cooperation.

### 2.2. Principle 2: An Explicit Microeconomic Model of Interactions.

#### Punishment in optional public good games.

Cooperation in large groups is a fundamental feature of human cooperation. In *n*-player social dilemmas, one possibility to stabilize cooperation is peer punishment (57). However, the emergence of peer punishment has been subject to a lot of debate and has been addressed by a number of evolutionary models (33, 58, 59). One possibility for the emergence of peer punishment is the option to abstain from a public good (33, 60). This means that agents have the choice to not participate in the collective endeavor, guaranteeing a payoff that is more than what everyone gets when no one contributes to the public good, but less than the payoff from widespread cooperation.

The standard model starts by considering a public goods game. Each individual can cooperate, by contributing a quantity *c* to a common pool; or defect by not contributing anything to the pool. The contributions in the pool are multiplied by a factor *r*, and the total amount is equally distributed among all distributed among *n* members of the group (or among all other players only (61)). Defection is the dominant strategy. In a second stage of the game, individuals are given the opportunity to punish defectors, at a cost *γ*, imposing a fine *β* to those in the group that did not contribute. Defectors are prevalent when the public goods game is compulsory (33).

**Fig. 2.**
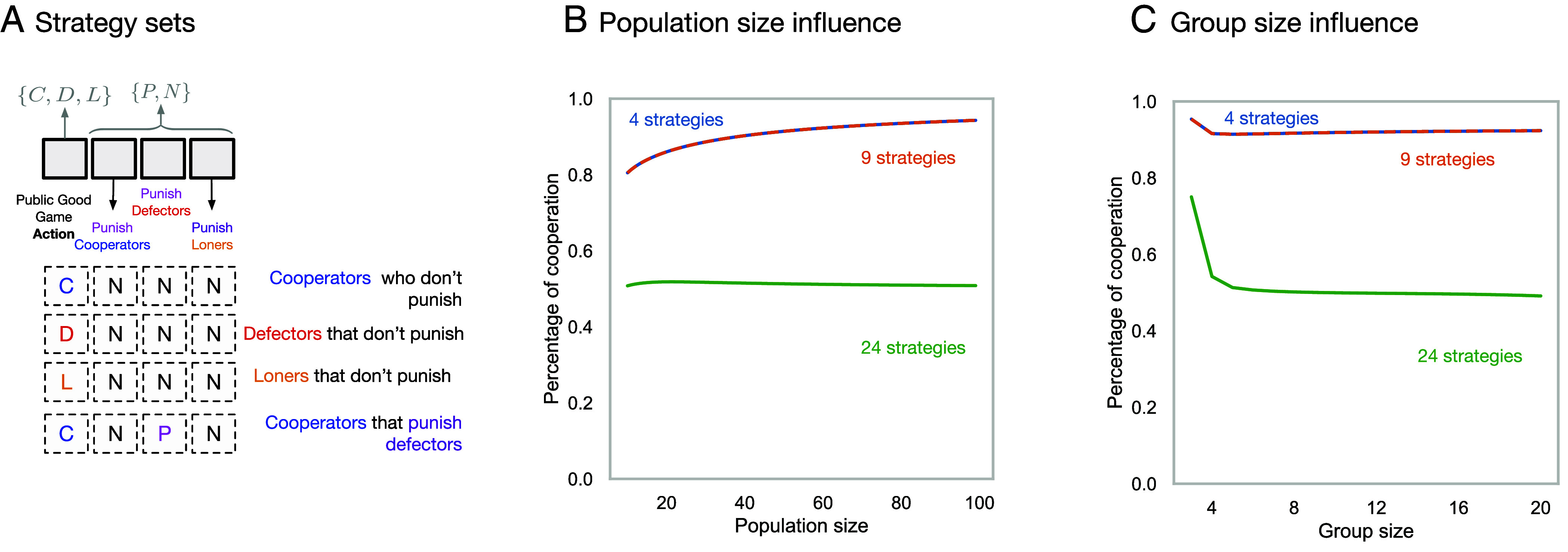
Strategy spaces in a public goods game with punishment. (*A*) Strategies in public good games with punishment can be conceived as a string with four elements. The first element contains the behavior of agents in the first stage of the game—they can cooperate or defect or abstain from playing the game. The second, third, and fourth elements are binary and determine whether the strategy punishes cooperators, defectors, or loners, respectively. The complete set of strategies contains $24=3\times 2^{3}$ strategies. Different subsets correspond to different microeconomic assumptions in the game. For example, the assumption that loners cannot be punished reduces the set to 12 strategies. Panels *B* and *C* show the average percentage of cooperation reached in the long-term for three different choices of strategy set in an imitation process, depending on population size and group size. The original set with 4 strategies has been introduced by Hauert et al. (33). The set with 9 strategies allows punishment to and from everyone except Loners (34). The set with 24 strategies follows Rand and Nowak (35), allowing for everyone to punish and be punished. Here, the sets with 9 and 4 strategies have the same abundance for strong selection, suggesting a robustness of the 4 strategy set to the extension to 9 strategies, but not to 24 strategies (parameters *r* = 3.0, *c* = 1.0, *σ* = 1.0, *β* = 1.0, *γ* = 1.0, *n* = 5, population size is *N* = 100, intensity of selection 1.0).

The public goods game can be made optional by introducing a “loner” strategy (62). A loner does not take part in the public endeavor, instead getting a guaranteed payoff *σ*. The public goods game is only played when there are two or more participants—otherwise all players get the loner payoff *σ*. In this system, we have now defined four strategies: Cooperators, Punishers that cooperate and punish defectors, Defectors, and Loners. The game parameters are usually chosen such that 0<σ<(r−1)c. This implies the risky collective endeavor pays off when everyone cooperates, but a payoff larger than that of universal defection is available via nonparticipation. As a result, loners offer a way out of defection and into cooperators and punisher. Overall, the dynamics lead to cycles of punishment-supported cooperation and defection. Punishment is the most abundant strategy in the long-term—leading to large levels of cooperation (Fig. 2).

#### Who can punish and who can be punished?.

This strategy set of four strategies (33) is biased. Cooperative strategies are twice as abundant as noncooperative ones under neutrality. This is partly because the original intent of the model was to explain pro-social punishment, but restricting punishment to only defectors not only does ignore computationally equivalent strategies but also does not take into account the empirical evidence that punishment can be used in an antisocial way, i.e., defectors punishing cooperators (63).

Detaching punishments from actions leads to 24 possible strategies, as described in Fig. 2*A*. This set is studied in ref. 35 and predicts the collapse of cooperation. But this prediction only holds if loners can be punished by those participating in the game (34). When loners are no longer punished or able to punish others, the original predictions from ref. (33) remain. This can be seen in Fig. 2, which depicts cooperation as a function of population (*A*) and group size (*B*). The restrictions on strategies are described in Table 1.

**Table 1.** Different restrictions on the strategy set arise from different microeconomic assumptions

Number ofstrategies	Restrictions	Ref.
4	Only cooperators can punish only defectors	(33)
9	Loners cannot punish or be punished	(34)
24	Everyone can punish and be punished	(35)

A strict assumption leading to four strategies that only cooperators can punish and punishment can only be directed at defectors. A more systematic assumption would be to assume that loners not taking part in the game are unable to monitor public good contributions and thus cannot punish those that take part in the game or be punished, leading to nine strategies. Finally, we could assume that no such restrictions apply, leading to 24 strategies.

From these models, we can infer that the microeconomic model for the interactions can be crucial and drive the outcome. Let us suppose that the public goods game represents hunting for food as a group. If loners are unable to monitor the contributions of others, it stands to reason that they are not able to punish. Should loners be punished? This in turn will depend on the specific situation that the game is modeling. A game is a model in itself, and spelling out the microeconomic assumptions behind can reveal which set of the strategy space is meaningful.

### 2.3. Principle 3: Connection to stylized facts.

#### Exogenous norms of reciprocity.

Indirect recipro city means agents rely on reputations when deciding to cooperate. The cost of cooperation can be offset by the benefits of accruing a good reputation. This mechanism is thought to explain important features of human cooperation, as humans are known to cooperate even in anonymous interactions.

**Fig. 3.**
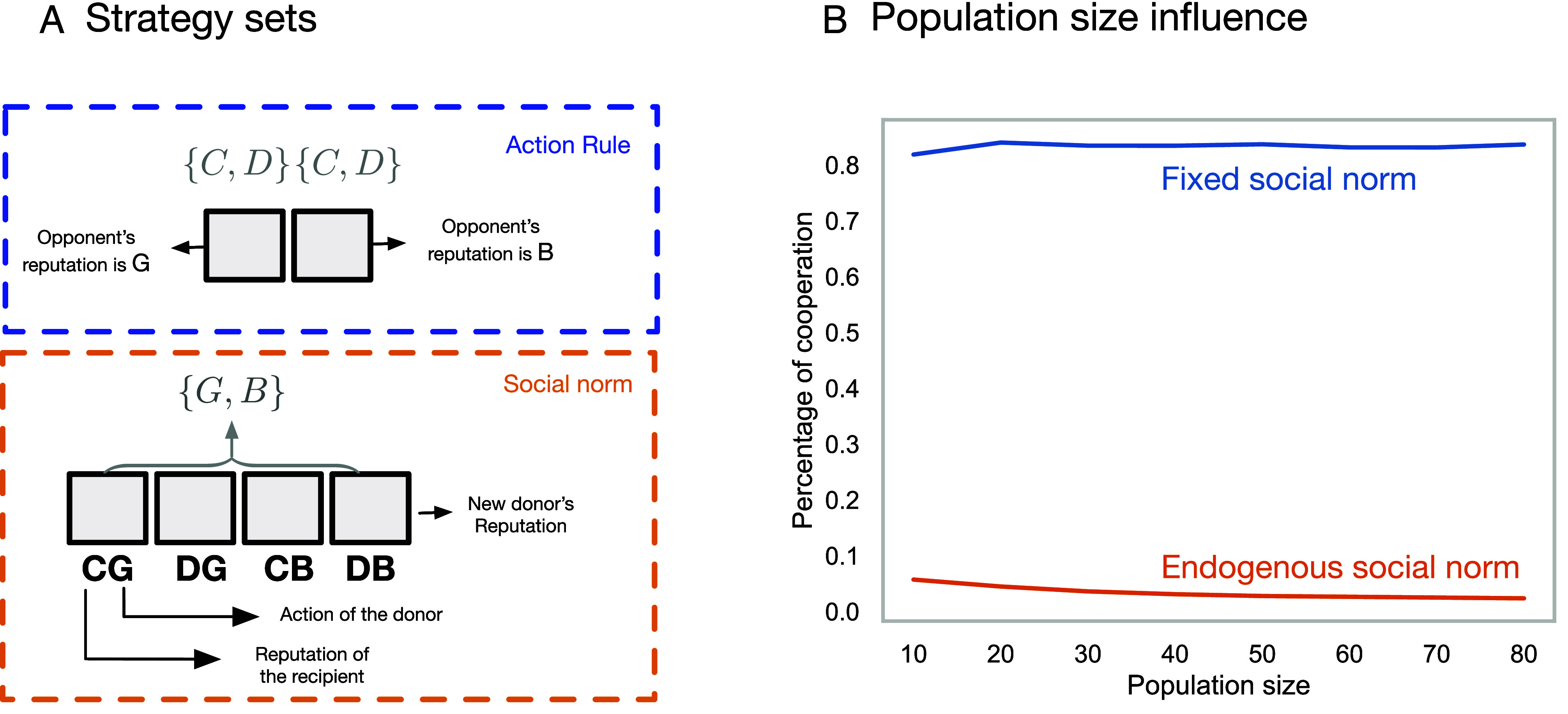
Strategy spaces in indirect reciprocity. (*A*) In games of reciprocity with binary reputations, strategies contain action rules and social norms. In standard models, social norms are exogenous and homogeneous, despite experimental evidence of norm variation across individuals. (*B*) Average percentage of cooperation reached in the long-term for two different sets of strategies. Exogenous norms assume one highly cooperative norm [“stern judging” (64)], thus agents only choose their Action Rules, i.e., how to react to the reputations of others (4 strategies). Endogenous norms let agents choose a combination of four Actions Rules as well as a Social Norm to update the reputations of others (4 action rules × 16 social norms = 64 strategies (population size is set to 50 and all other parameters follow ref. 65).

In standard models of indirect reciprocity, agents learn how to react to the reputations of others when playing a donation game (a particular kind of prisoner’s dilemma). For binary reputations, there are four possible action rules: Cooperators, Discriminators who only cooperate with those that have a good reputation; Antidiscriminators who only cooperate with those that have a bad reputation, and defectors. After each interaction, the reputation of a focal agent (usually called the donor) is updated according to a reputation norm—a function that produces a reputation value given the specific details of an interaction. The payoffs of the agents are derived from a series of interactions with opponents chosen at random. Action rules and social norms are described in Fig. 3*A*.

A recent review on the vast literature of indirect reciprocity can be found in ref. 67. Primarily, the literature has considered understanding which social norms stabilize cooperation. First, formulating specific social norms that can lead to cooperation (68), and later on defining a space of social norms and inspecting which norms can stabilize cooperation, e.g., refs. 69–71. More recent work has focused on analyzing the effect of stochastic dynamics and group size (64), as well the complexity of social norms (72, 73) and continuous reputations (74). Another line of work has focused on understanding what happens when reputations are private, although this begets the question of how close this model is to traditional models of direct reciprocity where no information is social (75, 76)—conceptually, indirect reciprocity has been centered around the idea that individuals do not only use their personal experience to decide who they cooperate with (77).

#### Endogenous norms of reciprocity.

While experimental studies of cooperation abound, specific experiments about indirect reciprocity are scarce (77–80), ref. 80 presents an experiment that sets to understand whether and when do people use contextual information, such as what was the reputation of the agent you helped—second-order information. This study finds that there is substantial variation in the social norms that people use. A natural question is what happens when social norms are part of the agents’ strategies and are allowed to evolve, together with the action rules? Xu et al. (65) extend the framework introduced by Santos et al. (72) to allow for endogenous social norms. The result is shown in Fig. 3*B*. Cooperation collapses due to neutral drift arising between agents that play the same strategy with different social norms. The payoff between them remains the same, paving the way for norms to cycle regularly in the population. Simulation models confirm that this lack of cooperation also holds for more complex norms (66).

This is not to say that models with endogenous norms are not useful. But the interplay between experimental work and theoretical models is important, particularly when the foundational models of cooperation are well understood. Models with connections to stylized facts are more likely to enlighten the field beyond adding to the list of potential mechanisms of cooperation.

## 3. Cooperative AI

The problem of cooperation has recently gained traction in the field of AI (18, 81). This is in part due to the advent of multiagent systems applications (82), and the unparalleled success of artificial agents in purely competitive games, such as board games and video games (83–85). While this interest is not completely new—see for example refs. 56, 86, and 87—recent AI advances in complex environments as well as the rise of personal AI assistants and distributed devices across a range of applications have made the topic of cooperation and artificial agents salient and relevant (88–90).

Reinforcement learning (RL) (91)—and its particular variant implemented with deep neural networks (92)—is one of the prominent techniques in this space. This family of algorithms is concerned with how agents can sequentially take actions in an environment in order to maximize some well-defined long-term reward. As a result, artificial RL agents in settings without centralized control are for the most part subject to the pitfalls of cooperation. Groups of agents who can work together have therefore difficulties in discovering policies (or strategies) that take advantage from cooperation (90, 93). This is problematic, because when artificial agents have the potential to interact with each other autonomously, they may not be equipped to take advantage of cooperation opportunities.

Multiagent RL (MARL) extends the single-agent RL framework to scenarios involving multiple agents. In RL, agents take actions in an environment in order to maximize a well-defined cumulative reward. As such, the following elements need to be precisely defined: i) A state space that describes all possible situations in which agents can find themselves. ii) An action space that describes all possible moves the agent can make in any of the previously defined states. iii) A reward structure that provides feedback from the environment, associating combinations of states and actions to rewards that can guide learning. In standard RL applications without multiple agents, the environments are such that the probabilities of transitions between states and the rewards associated with those transitions remain constant over time. This is known as stationarity and it means that the rules governing the environment and the outcomes of actions do not change as the agent interacts with the environment. A scenario with multiple agents learning simultaneously typically breaks this property, and thus specific techniques are developed to deal with the more complex setting, including techniques to detect and adapt to changes in the environment’s dynamics, which in multiagent settings typically involves understanding or predicting what opponents may do. This is in addition to the traditional challenge of RL that aims to balance exploration and exploitation.

Hyperparameters in RL are the configuration settings that are external to the model. They can significantly affect the performance and efficiency of an RL agent. These hyperparameters need to be defined before training begins and are often tuned to optimize the agent’s learning. Thus, while a rigorous definition of actions, states and rewards can lead to policies that are unbiased, the process of learning often relies on tuning a number of parameters that are often unrelated to the model itself.

### 3.1. MARL and Cooperation.

From the perspective of cooperation research, the recent literature on Cooperative AI can tackle two related, but distinct questions.

i) How does cooperation emerge in a system of selfish agents (that learn using the same algorithm)? We call this **the emergence problem** (e.g., ref. 89).
ii) How to best design an artificial agent that can learn to cooperate in environments with other (possibly distinct) agents? We call this **the design problem** (e.g., ref. 94).

We argue that the role that EGT can play in informing AI, and the role that AI can play in enriching cooperation research heavily depends on the question being asked. We now discuss how current challenges in the cooperation literature can intersect with these two questions.

The emergence question relates to understanding what is required for a group of (learning) agents to collectively achieve cooperation. This is essentially the same question asked traditionally in the context of the EGT cooperation literature. It is typically assumed that agents are homogeneous in the way in which they learn. This is the case in most studies around the evolution of cooperation, or social learning and cooperation. As such, one can expect similar answers to the question. Fundamental mechanisms that align individual and group incentives at the level of payoffs should be expected to work regardless of how agents learn.

However, there are potential synergies between these two areas of research (95–97). One popular setting is the harvest game, where mobile agents harvest a resource, but individual interests to harvest as much as possible are at odds with the group’s interest not to overexploit the resource; see, e.g., ref. 21. This game is reminiscent of models in evolutionary ecology, where food items arrive randomly at different rates in different patches and that foragers choose their patch such that each gets an equal share of resources. Biologists have developed theoretical models for this, abstracting from the movement of agents, differences between agents and many other issues—ultimately showing that there is an “ideal free distribution” of individuals into patches (22, 23). Despite these abstractions, the theoretical predictions were confirmed empirically in long-term observation studies of natural fish populations (24) and in controlled experiments in fish (25).

This high level of abstraction is typical for EGT, where scientists focus on abstract models that describe large populations with only a few different types of individuals—the focus is on strategic complexity, and not necessarily the adjacent complexities of the environment. Ultimately, this difference between environmental and strategic complexity is key to how researchers in the field of cooperation can benefit from AI models.

### 3.2. Richer Models Via AI?

The methodology in AI is of potential interest to those that study cooperation, because it can explore large policy spaces in games that are more complex. This can be useful for cooperation researchers who want to bring the theory of cooperation to more complex scenarios. In this case, we think the principles described above still apply. We next describe how each of these principles intersects with the methodology arising from AI.

#### Unbiased and computationally equivalent strategies.

The methodology in AI would typically lead to an unbiased and computationally equivalent set of strategies. This is because policies as discovered by RL arise from a description of fundamental states and actions—often the search space is so large that a complete enumeration of policies is not feasible. This methodology is useful in EGT, even if the space of strategies or policies is small. As shown above, considering all possibilities when defining a strategy space is desirable.

#### An explicit microeconomic model of interactions.

In AI applications, the environment is usually very explicit and rich—agents can typically move around in a spatial environment and interact with objects or other agents in that space. The view in which policies respond to environmental states ensures the environment is well defined, and actions that result in environmental changes in turn guarantee a sound microeconomic description. We want to emphasize that this contrasts with the traditional view from EGT, which relies on describing simpler games or payoff functions—but as the example of Axelrod’s tournaments shows, the environment can still determine the outcome, as it defines the mode of selection. While these sometimes leave room for interpreting what strategies are valid (Section 2.2), in EGT the primary interest is in the complexity arising from strategic interactions and incentives, and not necessarily in the environmental complexity per se. This is a balance that needs to be navigated in the literature. The complexity of an agent whose policies force them to be explicit about how they move in space is not necessarily going to shed some light on the fundamentals of cooperation. Perhaps RL techniques can be most fruitful in this question if they are combined with techniques of evolutionary dynamics—simple models of complex settings are still very valuable.

#### Connection to stylized facts.

Discussing the emergence of cooperation as a model of human agents, connections to empirical facts from the social sciences are still important in helping us focus on explaining stylized empirical features of cooperation. To argue that many additional models of cooperation are necessary to push the field forward — especially in areas where the connection to stylized facts is weak — is problematic. There is a well-known repertoire of general explanations for how cooperation can flourish in principle (1). The open question is how these mechanisms come together to explain the specificities of human cooperation (13). The nature of this question does not change whether we are using AI-inspired or more traditional methods.

### 3.3. AI and the Agent Design Question.

In pure AI applications, it is sometimes important to understand how we can design agents that can reap the benefits of cooperation, even if they are primarily driven by selfish rewards. This is a different question because we are not interested per se in creating or understanding how homogenous agents can learn to cooperate with each other. Instead, designing and agent that can reap the benefits of cooperation requires the design to be able to withstand other agents that may not cooperate or use a different algorithm.

Consider an agent whose intrinsic rewards allow them to identify cooperation opportunities (e.g., ref. 94). This agent needs to be able to effectively cooperate with other agents endowed with a different utility function. Assuming all agents in the system will have the same intrinsic reward corresponds to some level of central control that enforces utility functions (98)—in which case, cooperation could also just be centrally mandated.

It is conceivable that what we know about the emergence of cooperation can inform the design of agents artificial agents that can learn to cooperate. Recalling that certain prerequisites are necessary can guide the design of cooperative agents: “for direct reciprocity you need a face (=recognition), for indirect reciprocity you need a name (=communication)” (99). Crucially, these agents need to be resilient in a way that is usually not accounted for when discussing mechanisms of cooperation—an agent should be able to exploit cooperation opportunities against naive agents, while avoiding the exploitation from selfish agents—but in the cooperation literature, the set of agents is usually consisting of a small number of strategic types.

Methodologically speaking, the work in this area relies heavily on large-scale simulations, evolutionary dynamics can be used to analyze limiting cases that allow to gauge and validate the model (100).

## 4. Discussion

McNamara called for a change in EGT toward richer models that include important aspects of reality, such as between-individual variation, the ecological and life-history context of the situation, or the traits that are under selection (104). While we fully agree with these ambitions, our present proposal for a new generation of models is fundamentally different: a rich model in the sense of McNamara, which includes more aspects of reality, could still be biased in the choice of strategies. And a model with a complete strategy space could still remain abstract and not include relevant aspects of reality. However, as an important part of the new generation of models we envision is computation, it would be easier to include additional features into them and potentially limit the analytical analysis to a simplified version of the model.

There are many possible objections to our approach. For example, one could criticize the inclusion of behaviors that are not found to be relevant in reality, such as seemingly antisocial punishment strategies or behaviors in repeated games that do not induce any cooperation. However, how do we know that these are not relevant? Even if not abundant, such strategies can still influence the evolutionary dynamics, e.g., by providing paths out of—or into—undesired states. We suggest that evolutionary dynamics would be a perfect way to determine the relevance of such strategies.

One important thing to consider is the purpose of this kind of models. While we typically aim to explain the evolution of certain traits, an important issue is why similar traits of the same complexity do not evolve. We can develop a model for pro-social punishment, but such a model may become more powerful if it can explain the absence of antisocial punishment at the same time. Ultimately, it may be more interesting to know why some traits evolve while other ones do not than to only explain what is observed.

## Data Availability

Code and data have been deposited in Anonymous git (https://anonymous.4open.science/r/egt_ai-502D/).
